# Importation and Circulation of Vaccine-Derived Poliovirus Serotype 2, Senegal, 2020–2021

**DOI:** 10.3201/eid2810.220847

**Published:** 2022-10

**Authors:** Martin Faye, Ousmane Kébé, Boly Diop, NDack Ndiaye, Annick Dosseh, Abdoulaye Sam, Aliou Diallo, Hamet Dia, Jean Pierre Diallo, Ndongo Dia, Davy Evrard Kiori, Ousmane Madiagne Diop, Amadou Alpha Sall, Ousmane Faye

**Affiliations:** Institut Pasteur de Dakar, Dakar, Senegal (M. Faye, O. Kébé, H. Dia, N. Dia, D.E. Kiori, A.A. Sall, O. Faye);; Ministry of Health and Social Actions, Dakar (B. Diop, A. Sam, J.P. Diallo);; World Health Organization Inter-Country Support Team for Western Africa, Ouagadougou, Burkina Faso (A. Dosseh);; World Health Organization Senegal Country Office, Dakar (A. Diallo);; World Health Organization, Geneva, Switzerland (O.M. Diop)

**Keywords:** polio, vaccine-derived poliovirus serotype 2, viruses, environmental surveillance, acute flaccid paralysis, Senegal

## Abstract

Environmental surveillance for poliovirus is increasingly used in poliovirus eradication efforts as a supplement to acute flaccid paralysis (AFP) surveillance. Environmental surveillance was officially established in 2017 in Senegal, where no poliovirus had been detected since 2010. We tested sewage samples from 2 sites in Dakar monthly for polioviruses. We identified a vaccine-derived poliovirus serotype 2 on January 19, 2021, from a sample collected on December 24, 2020; by December 31, 2021, we had detected 70 vaccine-derived poliovirus serotype 2 isolates circulating in 7 of 14 regions in Senegal. Sources included 18 AFP cases, 20 direct contacts, 17 contacts in the community, and 15 sewage samples. Phylogenetic analysis revealed the circulation of 2 clusters and provided evidence on the virus introduction from Guinea. Because novel oral polio vaccine serotype 2 was used for response activities throughout Senegal, we recommend expanding environmental surveillance into other regions.

Human enteroviruses (HEVs) are ubiquitous and responsible for a spectrum of acute diseases in humans, including aseptic meningitis, encephalitis, acute flaccid paralysis, myocarditis, type 1 diabetes, and neonatal enteroviral sepsis through fecal-oral transmission (*1*,*2*). Belonging to the *Picornaviridae* family, HEVs are classified into 4 species (HEV-A, HEV-B, HEV-C, and HEV-D) covering >116 serotypes, including the polioviruses, members of the HEV-C species, which are divided into 3 serotypes named PV1, PV2, and PV3 (*3*).

The World Health Organization (WHO) recommended clinical surveillance of polioviruses by investigating cases of acute flaccid paralysis (AFP) in children <15 years of age, an age group considered high risk for infection. The last confirmed wild poliovirus (WPV) case in Senegal was reported in the Kaolack health district; the patient experienced AFP on April 30, 2010. The last supplementary immunization activities using the trivalent oral polio vaccine (OPV) took place in April 2016 in the Dakar region after identification of an ambiguous vaccine-derived poliovirus (VDPV) serotype 2 (aVDPV2) (*4*).

The Global Polio Eradication Initiative (GPEI) has established the testing of environmental or raw sewage samples to detect VDPV and WPV to complement AFP surveillance in children <15 years of age in polio-free countries in Africa (*5*–*7*). The reason for this surveillance is that 99% of poliovirus infections are asymptomatic and therefore not detected by AFP surveillance, but the virus is shed for weeks in the feces of infected persons. Environmental surveillance has played a pivotal role in detecting VDPVs in raw sewage samples and has provided meaningful data on the presence of polioviruses in communities (*7*,*8*). As of October 2021, the GPEI has initiated environmental surveillance in 309 sites from 36 countries (*6*,*9*,*10*), which enabled the identification of polioviruses in sewage and helped WHO to initiate investigations in the communities and confirm an outbreak of VDPV serotype 2 (VDPV2) in a low vaccination coverage area (*11*).

In Senegal, scarce data were collected during 2007–2015 through surveillance projects carried out at Institut Pasteur de Dakar (*12*). In 2017, environmental surveillance for polioviruses was initiated as part of a collaboration between the Prevention Office at the Senegalese Ministry of Health and Social Action (MoHSAS), the Dakar Medical Region, Institut Pasteur de Dakar, the National Office of Sanitization in Senegal, and WHO. Sewage samples were collected monthly from 2 sites located in the city of Dakar (Khourounar lifting station and Cambérène sewage treatment site). A circulating VDPV2 (cVDPV2) isolate was detected in a sewage sample collected at the Khourounar site on December 24, 2020 (*13*). Data received from the reference sequencing laboratory for polio at the US Centers for Disease Control and Prevention (Atlanta, GA, USA) suggested an introduction of the cVDPV2 from the Ratoma district in Guinea (*13*). This notification alerted the MoHSAS to the occurrence of a poliomyelitis outbreak caused by cVDPV2; investigations were initiated in the communities.

We describe the data from a 7-year AFP and environmental surveillance program in Senegal for early detection of cVDPV2 and provide insights into virus circulation through the country since December 2020. As part of the GPEI, our study did not directly involve human participants but included stool samples and cell-culture isolates from AFP cases collected as part of routine surveillance for polio for public health purposes in Senegal. WHO and the national ethical committee at the Ministry of Health and Social Actions approved and supervised our study, considering all applicable national regulations governing the protection of human subjects. We obtained cleared oral consent from all patients or their parents or relatives.

## Methods

### Stool Sample Collection

In Senegal, AFP cases are ascertained either by surveillance focal points in medical districts that send notification to the Epidemiologic Surveillance Unit at MoHSAS within 72 hours of identification (*4*). As recommended by WHO, we collected 2 fecal specimens for laboratory investigations >24 hours apart and <14 days from the onset of paralysis from cases of AFP to identify the causative enterovirus agent. Specimens were tested in the intercountry WHO-accredited laboratory at Institut Pasteur de Dakar. We reported laboratory data from AFP cases in children to MoHSAS and WHO country and African Region (AFRO) offices each week.

### Sewage Sample Collection

We collected sewage samples monthly from 2 sites by grab method as described in WHO guidelines for environmental surveillance (*11*,*12*,*14*). In brief, we collected 1 liter of sewage sample using a bucket, with strict compliance to safety requirements. We transferred sewage samples to the laboratory within 2–4 hours under cold chain container maintained at 4°C and transported to the WHO Intercountry Reference Laboratory for Poliomyelitis at Institut Pasteur de Dakar for processing. We reported data to MoHSAS and the WHO country and AFRO offices each week.

### Sewage Sample Processing

We processed sewage samples by polyethylene glycol precipitation method as previously described (*11*). We performed virus isolation using 3 flasks (25 cm^2^) of L20B and 2 flasks of rhabdomyosarcoma (RD) cell line, in accordance with standard WHO protocols (*15*,*16*). We monitored the inoculated cell lines for development of cytopathic effect (CPE) for 5 days. If CPE appeared in any cell line, we performed cross-passage to the opposite cell line. If no effect appeared, we performed a blind passage in the same cell line and monitored cells for cytopathic effect for 5 additional days. Samples with no CPE in both cell lines, even after the blind passage, were considered negative, whereas RD-positive and L20B-negative samples were classified as nonpolio enterovirus. We classified L20B-positive isolates as suspected poliovirus and subjected them to real-time reverse transcription-PCR using a qScript XLT qPCR Toughmix system kit (Quantabio, https://www.quantabio.com) with the intratypic differentiation kit (version 5.2) as recommended for identification of a poliovirus strain (*17*). The kit included primers for panenterovirus, panpoliovirus (pan-PV), Duplex wild poliovirus type 1 (WPV1), African wild poliovirus type 3 (AFR WPV3), and South Asian wild poliovirus type 3 (SOAS WPV3).

### Sequencing

We spotted the PV2-positive isolates on FTA cards and sent to the CDC reference sequencing laboratory for polio. Samples were later processed for high-throughput RNA sequencing; complete sequences of the poliovirus viral protein (VP) 1 genomic region, which contains a major neutralizing antibody binding site, were generated (*18*,*19*). The number of mutations within the VP1 region of the live attenuated OPV strain was determined; genetically divergent VDPV strains had been classified as cVDPV2. In addition, a phylogenetic analysis was conducted to determine the poliovirus sequence most likely related to the virus (*19*).

### Phylogenetic Tree Inference

We performed Bayesian phylogenetic analysis for estimation of data quality and selection of the best-fit nucleotide substitution model using ModelFinder test (*20*) on the IQ-TREE version 1.6.3 web server (http://iqtree.cibiv.univie.ac.at) (*21*)*.* We constructed a maximum-likelihood tree with sequences of VP1 using FastTree version 2.1.7 (*22*) with the best-fit nucleotide substitution model to our sequence dataset. We labeled nodes with local support values, which were computed with the Shimodaira-Hasegawa test (for 5,000 replications, and visualized the topology with FigTree version 1.4.2 (http://tree.bio.ed.ac.uk/software/figtree).

## Results

### Temporal Distribution of Isolated Enteroviruses

**Figure 1:**
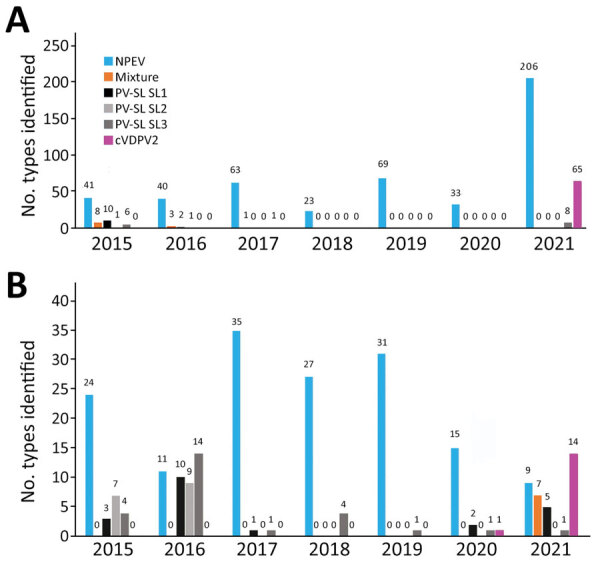
Temporal distribution of enterovirus isolates detected in Senegal during 2015–2021. A) Enteroviruses detected from acute flaccid paralysis surveillance, including all human specimens (cases, close contacts, and community contacts). B) Enteroviruses detected from environmental surveillance. cVDPV2, circulating vaccine-derived poliovirus serotype 2; NPEV, nonpolio enterovirus; PV-SL, Sabin-like poliovirus.

During 2015–2021, we received a total of 2,971 stool samples and 281 sewage samples from the AFP and environmental surveillance in Senegal. The AFP surveillance includes all human specimens (AFP cases, close contacts, and community contacts). No wild or VDPV serotype 1 or 3 were isolated in Senegal during this surveillance period; 1 VDPV2 was isolated from a sewage sample collected on December 24, 2020 and later classified as cVDPV2. In 2021, we identified a total of 79 cVDPV2-positive isolates in 14 laboratory-confirmed sewage samples and 65 stool samples from 18 AFP cases, 20 direct contacts, and 17 contacts in the community. During this 7-year surveillance period, we detected a total of 28 Sabin-like (SL) polioviruses (12 SL1, 2 SL2, and 15 SL3) from AFP surveillance and 63 (21 SL1, 16 SL2 and 26 SL3) from environmental surveillance (Appendix Table). We recorded the highest prevalence of SL polioviruses in 2015–2016, with a total of 17 isolates from AFP surveillance in 2015 and 33 isolates from environmental surveillance in 2016. In addition, we detected 469 nonpolio enterovirus from AFP surveillance and 152 from environmental surveillance. Of interest, the highest number of nonpolio enterovirus isolates, 206, was identified from AFP surveillance in 2021. We detected 12 mixtures from AFP surveillance during 2015–2017 and 7 from environmental surveillance in 2021 (Figure 1). No homotypic mixture has been found. However, from AFP surveillance, we detected 2 trivalent mixtures (SL1+SL2+SL3) in 2015–2016 and detected a total of 10 bivalent mixtures (5 SL1+SL2 and 2 SL1+SL3 in 2015, 2 SL1+SL2 in 2016, and 1 SL1+SL3 in 2017). Seven bivalent mixtures (5 SL1+cVDPV2, 1 SL1+SL3, and 1 SL3+cVDPV2) were detected from environmental surveillance.

### Geographic Distribution of cVDPV2 in Senegal

As of December 31, 2021, a total of 70 cVDPV2 sequences were recorded from Senegal, including 18 AFP cases, 20 direct contacts, 17 contacts in the community, and 15 from sewage samples. One of the positive sewage samples collected in the Dakar region on May 6, 2021, was a mixture of cVDPV2 and VDPV1. The VDPV1 from Senegal has 10 nt differences from the VP1 sequence of Sabin 1 and was not genetically linked to any previously sequenced VDPV1s (data not shown). We identified cVDPV2 in 7 of 14 regions in Senegal: 24 isolates from Diourbel, 17 from Dakar, 11 from Thiès, 8 from Fatick, 6 from Matam, 2 from Louga, and 2 from Kaolack (Appendix Figure).

### Phylogenetic Analyses

**Figure 2:**
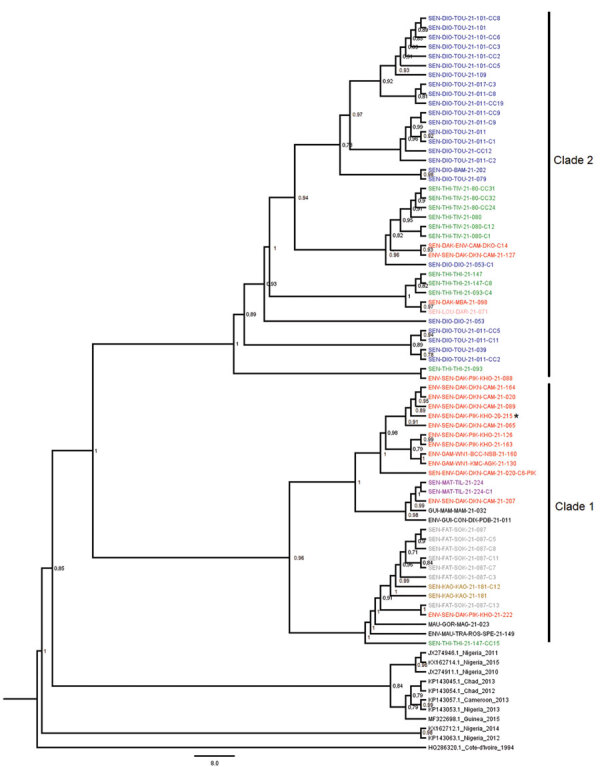
Maximum-likelihood tree based on complete viral protein 1 sequences of cVDPV2 isolates circulating in Senegal during 2020–2021. The tree is midpoint-rooted; nodes are labeled with local support values computed using the Shimodaira-Hasegawa test for 5,000 bootstrap replications. Strain identifiers are designated as follows: SEN-XXX-XXX-21-xxx indicates an isolate from an acute flaccid paralysis (AFP) cases; SEN-XXX-XXX-21-xxx-Cx, close contact of an AFP case; SEN-XXX-XXX-21-xxx-CCxx, community contact of an AFP case; ENV-XXX-XXX-XXX-XXX-21-xxx, isolate from a sewage sample; SEN-ENV-XXX-XXX-XXX-21-Cx-XXX, community contact around a positive environmental site. Isolate names are color-coded as follows: dark blue, new characterized isolates from the Diourbel region (SEN-DIO); green, the Thiès region (SEN-THI); red, the Dakar region (SEN-DAK); pink, the Louga region (SEN-LOU); purple, the Matam region (SEN-MAT); gray, the Fatick region (SEN-FAK); brown, the Kaolack region (SEN-KAO); black, Guinea (GUI), Mauritania (MAU), and The Gambia (GAM), and previous sequences of cVDPV2 from West Africa countries. Asterisk * indicates the first sequence isolated from sewage in Senegal in December 2020. cVDPV2, circulating vaccine-derived poliovirus serotype 2.

We submitted sequences to GenBank (accession nos. ON604861–950). The general time-reversible with a gamma distribution of 4 categories rate was the best nucleotide substitution model for our sequences dataset. We inferred maximum-likelihood trees in FastTree version 2.1.7 (*22*); phylogenetic analysis revealed that these new characterized cVDPV2 sequences from Senegal belonged to the NIE-JIS poliovirus lineage, which emerged from Nigeria in 2005 (*23*) and clustered closely with isolates sampled in neighboring countries such as Guinea, The Gambia, and Mauritania in 2021 (Figure 2). 

Since its first introduction into Senegal in December 2020, VDPV has separated into 2 phylogenetic clades. Clade 1 comprised only the isolates from Dakar, Thiès, Fatick, Kaolack, and Matam. However, clade 2 comprised sequences from Dakar, Diourbel, Thiès, and Louga and grouped with isolates from Guinea, Mauritania, and The Gambia. Of interest, sequences from Mauritania were related to the isolates from the Thiès region, and sequences from The Gambia were related to the isolates from the Dakar region, whereas the isolates from Guinea emerged before those from the Dakar and Matam regions. The phylogenetic data confirm the epidemiologic data received from the CDC reference sequencing laboratory for polio regarding the introduction of cVDPV2 in Senegal from Guinea and the virus spread from Senegal to The Gambia (Figure 2).

## Discussion

WHO has included environmental surveillance as a supplement to AFP surveillance in the strategic plan of the Global Polio Eradication Initiative. Examining sewage samples has been shown to be a more sensitive method to detect low-level circulation of WPV and VDPV; combining this environmental surveillance for polioviruses with enhanced AFP surveillance is expected to support eradication (*7*,*9*).

Excretion of poliovirus may contaminate surface-water sources for drinking water, recreational activities, aquaculture, and irrigation. The amount of virus so excreted can reach 10^7^ infectious dose/day/person (*8*); such an environment could lead to reintroduction of poliovirus in certified polio-free areas, especially in population groups with low immunization coverage. Oral poliovirus vaccine strains, VDPV, and even WPV strains may remain infectious for as long as 2 months in sewage depending upon environmental factors, including inactivation by sunlight and high temperatures (*7*,*8*). Circulation of enteroviruses in sewage is a proven indicator of their presence in some communities (*5*,*8*). Environmental surveillance can provide valuable information on virus circulation or reintroduction, particularly in urban populations with no active surveillance (*6*,*7*,*9*).

Since 2017, several genetically-distinct cVDPV2 outbreaks continue to be reported across the WHO AFRO area; the virus emerged in 28 countries in Africa, including Senegal, during January 2020–June 2021 (*24*–*26*). Through GPEI programs, WHO has implemented outbreak response measures in 21 countries, including Senegal, in response to the ongoing cVDPV2 outbreak (*25*).

The detection of cVDPV2 from sewage in Senegal in December 2020 led to a major public health investigation that identified the source as a virus imported from Guinea. Thereafter, the virus was detected in sewage, in the community, and from AFP cases. The epidemiologic data showed that 2 additional independent introductions have been recorded during this 2020–2021 cVDPV2 outbreak and the virus circulated in 7 of the 14 regions in Senegal. The second introduction was reported on February 18, 2021, in the Diourbel region from Mali, and the third one was recorded in August 2, 2021, in the Thiès region from Côte d’Ivoire (*13*). In addition, epidemiologic data showed an evidence of virus spread from the Dakar region in Senegal to The Gambia in June 2021 and Bissau Guinea in October 2021 (data not shown).

Our data demonstrated not only the importance of environmental surveillance to complement AFP surveillance, but also its sensitivity to detect all serotypes of poliovirus, as previously reported (*7*,*27*). Mass vaccination using a vaccine based on the inactivated poliovirus vaccine (*28*) was ruled out as a public-health response in the Touba district, Diourbel region in June 2021 because no dose of the improved novel oral polio vaccine serotype 2 (nOPV2) was available in the country at that time (*29*). Fortunately, the immunizations done on a smaller scale have highly contributed to reduce the number of confirmed cVDPV2 cases in that region. However, the virus successively emerged in new places including the Dakar, Thiès, Fatick, Kaolack, Louga, and Matam regions; 2 mass vaccination response campaigns based on the nOPV2 that contains a genetically stabilized serotype 2 strain less prone to reversion (*30*) were implemented at country level on December 17–19, 2021, and February 25–29, 2022 (*31*). These 2 mass vaccination response campaigns based on the nOPV2 (*29*) have contributed to interrupt cVDPV2 transmission in Senegal since the last cVDPV2 confirmed contact was identified on November 19, 2021, in the Matam region (*13*). Because serotype 2 immunity could decline in the years following vaccination campaigns, further seroepidemiologic studies would provide a quantitative basis for supporting decisions on the magnitude of future vaccination response when new detections are identified, particularly in countries that do not have environmental surveillance program (*6*).

Environmental surveillance in Senegal is restricted to the Dakar region. The probable silent circulation of these cVDPV2 isolates for several months before the first isolation in Dakar in December 2020 could have been caused by the lack of surveillance in the other 13 regions, which had limitations, such as logistics issues, for sample transportation and difficulty finding convergent sewage effluent from sufficiently large catchment populations. Actions such as increasing the number of sewage collection sites, ensuring regular environmental testing and expanding the testing to other geographic regions, especially those with high incidence of polio circulation, would support investigation of cVDPV2 circulation in Senegal and monitor the rollout of nOPV2 (*6*).

The phylogenetic data have demonstrated virus introduction to Senegal from Guinea, likely caused by population movement across the borders. The virus spread within Senegal could have been caused by the absence of immunity against poliovirus serotype 2 in most children born after the global vaccine switch (*12*). The sequences generated in this study could serve in further phylodynamics studies and could include complete VP1 sequences from more West Africa countries to determine the circulation dynamics of the virus and the number of introduction events in each country during this cDVPV2 outbreak. They could be also useful in analyses targeting the exact period of divergence of these Senegal isolates from their most recent common ancestor. Sequences of cVDPV2 from other neighboring countries such as Mali, Bissau-Guinea, and Côte d’Ivoire are not publicly available at this time.

Because the international spread of VDPV continues to be a public health emergency of international concern, the GPEI should reinforce capacity of countries involved in both clinical and public health functions including surveillance, diagnosis, prevention, treatment, community immunization response, and research and health promotion targeting particularly the immunocompromised children, who can excrete the virus for several months, to limit its global transmission (*32*). Considering the introduction in Malawi and Mozambique of serotype 1 wild poliovirus imported from Pakistan (*33*,*34*), GPEI should implement urgent measures to forestall its spread such as routine immunization for members of the susceptible population who were missed or were only partially protected, to complement routine immunization and regular supplemental immunization activity in polio-free countries to prevent introduction of WPV and minimize the risk of circulating VDPV (*35*,*36*).

Appendix Additional information about circulation of vaccine-derived poliovirus serotype 2, Senegal, 2020–2021.
