# Supplementary material for: Importation and Circulation of Vaccine-Derived Poliovirus Serotype 2, Senegal, 2020–2021
Source: Emerg Infect Dis. 2022 Oct;28(10):2027–34. doi: 10.3201/eid2810.220847 (PMC9514370; doi:10.3201/eid2810.220847)
Supplement: Appendix — Additional information about circulation of vaccine-derived poliovirus serotype 2, Senegal, 2020–2021. [file 22-0847-Techapp-s1.pdf]

Article DOI: https://doi.org/10.3201/eid2810.220847

Importation and Circulation of Vaccine-Derived Poliovirus Serotype 2, Senegal, 2020–2021

Appendix

Appendix Table. Temporal distribution of enterovirus types identified through environmental surveillance and acute flaccid paralysis surveillance, Senegal, 2015–2021*

Virus type	2015	2016	2017	2018	2019	2020	2021	Total no. of EV type identified
Environmental surveillance								
NPEV	24	11	35	27	31	15	9	152
Mixture	0	0	0	0	0	0	7	7
PV-SL1	3	10	1	0	0	2	5	21
PV-SL2	7	9	0	0	0	0	0	16
PV-SL3	4	14	1	4	1	1	1	26
cVDPV2	0	0	0	0	0	1	14	15
Total no. received samples	40	45	50	50	50	24	22	281
AFP surveillance								
NPEV	41	40	63	23	69	33	200	469
Mixture	8	3	1	0	0	0	0	12
PV-SL1	10	2	0	0	0	0	0	12
PV-SL2	1	1	0	0	0	0	0	2
PV-SL3	6	0	1	0	0	0	7	14
cVDPV2	0	0	0	0	0	0	65	65
Total no. received samples	362	361	310	257	366	271	1,044	2,971

*AFP, acute flaccid paralysis; cVDPV, circulating VDPV2; EV, enterovirus; NPEV, nonpolio enterovirus; PV, poliovirus; SL, Sabin-like; VDPV, vaccine-derived poliovirus type 2.

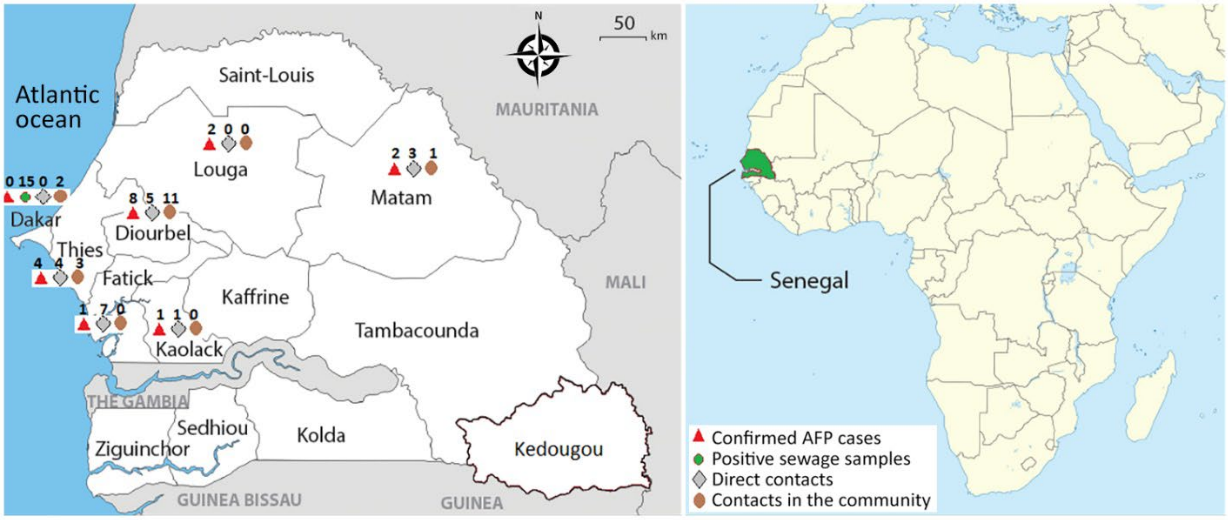

Appendix Figure. Geographic distribution of the cVDPV2 isolates identified in Senegal from December 2020 to December 2021. The total numbers of acute flaccid paralysis cases, direct contacts and contacts in the community detected in each region was highlighted in red, gray and brown, respectively. The total number of positive sewage samples detected in the Dakar region was color-coded in green.
